# Supplementary material for: Design of Novel Membranes for the Efficient Separation of Bee Alarm Pheromones in Portable Membrane Inlet Mass Spectrometric Systems
Source: Int J Mol Sci. 2024 Aug 7;25(16):8599. doi: 10.3390/ijms25168599 (PMC11354691; doi:10.3390/ijms25168599)
Supplement: Supplementary file 1 [file ijms-25-08599-s001.zip › ijms-3115845-supplementary.pdf]

Supplementary Materials

Design of Novel Membranes for the Efficient Separation of Bee Alarm Pheromones in Portable Membrane Inlet Mass Spectrometric Systems

[1]University of Novi Sad, Faculty of Sciences, Department of Physics, Trg D. Obradovića 4, 21000 Novi Sad, Serbia; stevan.armakovic@df.uns.ac.rs

[2]BioSense Institute, University of Novi Sad, Dr Zorana Djindjića 1, 21000 Novi Sad, Serbia; boris.brkic@biosense.rs, daria.ilic@biosense.rs

*Correspondence: stevan.armakovic@df.uns.ac.rs, boris.brkic@biosense.rs

Table S1. Names and optimized geometries of alarm bee pheromones considered in this work.

Molecule name	Optimized geometry
1-butanol	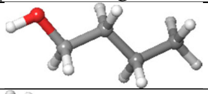
1-octanol	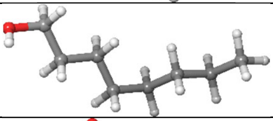
2-heptanone	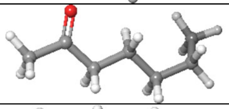
2-methyl-1-butanol	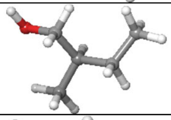
11-eicosen-1-ol	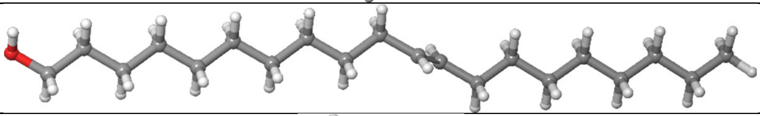
Furfural	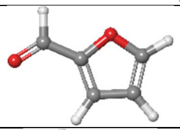
Acetamide	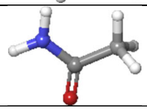
Ethyl oleate	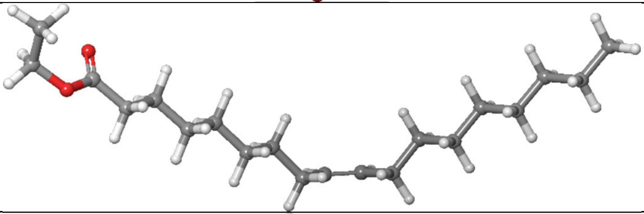
Isoamyl alcohol	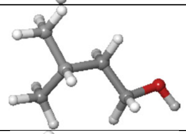
Propionic acid	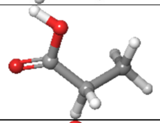
Isobutyiramide	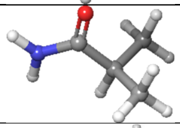
Isopentyl acetate	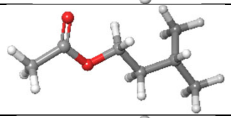
Isobutenyl carbinol	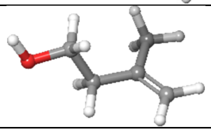
